# Geographic access to care is not a determinant of child mortality in a rural Kenyan setting with high health facility density

**DOI:** 10.1186/1471-2458-10-142

**Published:** 2010-03-17

**Authors:** Jennifer C Moïsi, Hellen Gatakaa, Abdisalan M Noor, Thomas N Williams, Evasius Bauni, Benjamin Tsofa, Orin S Levine, J Anthony G Scott

**Affiliations:** 1 Department of International Health, Johns Hopkins Bloomberg School of Public Health, Baltimore, MD, USA; 2 KEMRI/Wellcome Trust Research Programme, Kilifi, Kenya; 3 Malaria Public Health and Epidemiology Group, KEMRI/Wellcome Trust Research Programme, Nairobi, Kenya; 4 Nuffield Department of Clinical Medicine, John Radcliffe Hospital, Oxford, UK; 5 District Medical Officer for Health, Kilifi, Kenya

## Abstract

**Background:**

Policy-makers evaluating country progress towards the Millennium Development Goals also examine trends in health inequities. Distance to health facilities is a known determinant of health care utilization and may drive inequalities in health outcomes; we aimed to investigate its effects on childhood mortality.

**Methods:**

The Epidemiological and Demographic Surveillance System in Kilifi District, Kenya, collects data on vital events and migrations in a population of 220,000 people. We used Geographic Information Systems to estimate pedestrian and vehicular travel times to hospitals and vaccine clinics and developed proportional-hazards models to evaluate the effects of travel time on mortality hazard in children less than 5 years of age, accounting for sex, ethnic group, maternal education, migrant status, rainfall and calendar time.

**Results:**

In 2004-6, under-5 and under-1 mortality ratios were 65 and 46 per 1,000 live-births, respectively. Median pedestrian and vehicular travel times to hospital were 193 min (inter-quartile range: 125-267) and 49 min (32-72); analogous values for vaccine clinics were 47 (25-73) and 26 min (13-40). Infant and under-5 mortality varied two-fold across geographic locations, ranging from 34.5 to 61.9 per 1000 child-years and 8.8 to 18.1 per 1000, respectively. However, distance to health facilities was not associated with mortality. Hazard Ratios (HR) were 0.99 (95% CI 0.95-1.04) per hour and 1.01 (95% CI 0.95-1.08) per half-hour of pedestrian and vehicular travel to hospital, respectively, and 1.00 (95% CI 0.99-1.04) and 0.97 (95% CI 0.92-1.05) per quarter-hour of pedestrian and vehicular travel to vaccine clinics in children <5 years of age.

**Conclusions:**

Significant spatial variations in mortality were observed across the area, but were not correlated with distance to health facilities. We conclude that given the present density of health facilities in Kenya, geographic access to curative services does not influence population-level mortality.

## Background

In the 1970s, the incipient Primary Health Care movement brought the international community's attention to the specific health issues facing the poorest members of developing country societies. The "Health for All" doctrine, crystallized at the Alma Ata conference in 1978, advocated for community-based interventions to address health inequalities and the health of the poor, with the over-arching goal of combating health inequities [1]. The late 1990s witnessed renewed interest in equity in health [2,3]: the World Bank announced a new Health, Population and Nutrition strategy targeting the poor in 1997 [4] and the World Health Organization (WHO) initiated the Integrated Management of Childhood Illness program, which strives to enhance health care for all children in resource-poor settings [5].

In this context, the WHO supported studies to develop standardized methods for measuring inequalities and track changes resulting from health interventions. These studies focused primarily on disparities in health status and access to health services across socio-economic groups [6-10]. Our knowledge of geographic inequities in health is more limited and stems from country-wide analyses of Demographic and Health Survey data (DHS, Macro International Inc, Calverton, MD), which have shown higher mortality in certain provinces or in rural compared to urban areas [11]. At the micro level, distance to health facilities is known to predict care-seeking practices [12-19] but its impact on child survival has only been described in a small number of studies with inconsistent results [20-23].

The 1997 Kenyan health policy strategic framework states that all households should have access to health services within a 5 km range [24], in accordance with the notion that improved physical access could lessen delays in seeking care and time traveled to obtain treatment for childhood illnesses. Analyses of national health facilities databases showed that by 2003, this benchmark had been achieved for 82% of the population [25] and that by 2008, a 50% increase in the number of facilities - primarily within the government sector - had brought this estimate up to 89% [26]. In this study, we aimed to characterize spatial variations in child mortality in Kilifi District, Kenya, and evaluate the effect of distance to health facilities on child survival in a context of increased health services density.

## Methods

In this paper, we present an analysis of the data routinely collected by the Epidemiological and Demographic Surveillance System (Epi-DSS) in Kilifi District, Kenya, a member of the INDEPTH network of demographic surveillance sites.

### Study site

Kilifi District is a largely rural area situated on the Indian Ocean coast of Kenya, with a tropical climate characterized by two dry seasons and two rainy seasons each year. Kilifi District Hospital in Kilifi serves as a primary care center and first-level referral facility for the district. The KEMRI-Wellcome Trust Research Programme has performed hospital and field-based epidemiological research in Kilifi for two decades.

### Mortality data collection

The Epi-DSS covers approximately 900 km^2 ^around Kilifi District Hospital and was created in 2000 to track a population of over 220,000 people. After completion of the initial census in 2001, all homesteads in the Epi-DSS area were visited two to three times each year to collect information on births, deaths, in-migrations and out-migrations of household members. Beginning March 2003, pregnancy data was recorded to permit assessment of pregnancy outcomes and improved ascertainment of neonatal and early infant deaths.

### Mapping

**Figure 1:**
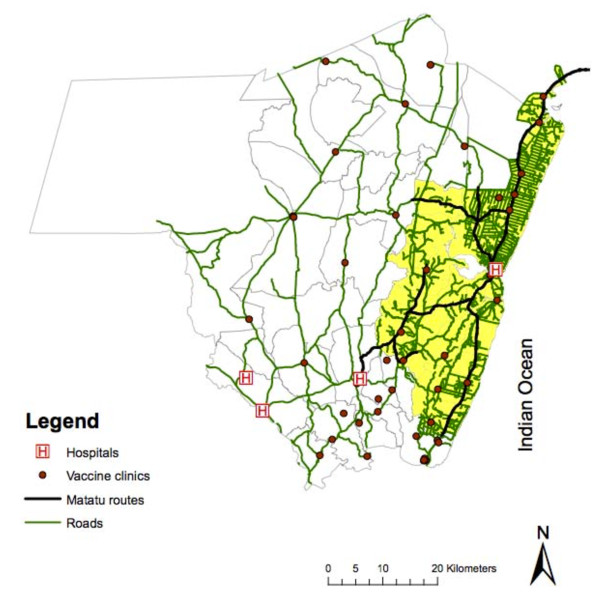
**Kilifi area health facilities and transport networks**.

The census area comprises 15 administrative locations, further divided into 40 sublocations. It has been mapped using Magellan (Magellan Navigation Inc, Santa Clara, CA) and e-Trex (Garmin Ltd, Olathe, KS) Geographic Positioning Systems technology, providing detailed information on topography, footpaths and roads, and human occupation of the area, including the coordinates of all homesteads. In January 2007, we collected data on the seven matatu (local bus) routes, including speed, frequency and cost of travel. One of these routes followed the only paved road in the Epi-DSS area, the Mombasa-Malindi coastal road. All geographic data were imported via Datasend, Map Source, or DNRGarmin software into ArcGIS 9.2 (ESRI, Redlands, CA) for mapping and analysis (Figure 1).

### Health facilities

A survey of health facilities in Kilifi District was conducted in September 2006. Four hospitals, 47 vaccine clinics (clinics offering childhood immunization among other preventive or curative services), and 100 other public, private or NGO-run health facilities were identified and mapped (Figure 1). Residents of the Epi-DSS may also access inpatient care outside the district, at Malindi District Hospital, which was therefore incorporated into our analysis.

### Cost-distance algorithm

Travel time to hospitals and vaccine clinics was calculated using an ArcGIS cost-distance algorithm. We divided the study area into 100-m × 100-m cells and created an impedance raster (grid) defining the speed of travel through each cell. The algorithm computes travel time from each health facility to all neighboring cells, then from these to all of their neighboring cells, proceeding iteratively until the entire area is covered. Thus, it delineates a catchment area for each health facility and obtains travel time to this facility from all cells in its catchment area. For pedestrian travel time, we assumed speeds of 5 km/hr on roads and footpaths and 2.5 km/hr off-road. In the vehicular model, matatu speeds were used on matatu routes and pedestrian speeds elsewhere. Kilifi Creek constitutes a natural barrier to travel and was attributed high impedance (1.25 km/hr speed). Changes in elevation in the Epi-DSS area are small and were not incorporated into the impedance raster.

### Data analysis

For each individual, we observed a series of dated, spatially-defined demographic events which were used to construct consecutive, non-overlapping observation periods. Each observation period was linked to residence in a homestead with known geographic coordinates. This data structure enabled us to perform survival analysis on a dynamic cohort of children entering and exiting risk sets over time.

We constructed Kaplan-Meier survival curves and instantaneous hazard curves by administrative location and by stratum of travel time to hospitals and vaccine clinics, as well as by sex, ethnic group (Giriama, Chonyi, Kauma, Duruma, Luo, Jibana, and "other" which combines all groups with <40 deaths), maternal education (proportion of women 15-49 years old with any education in a given sublocation: <0.5, 0.5-<0.6, 0.6-<0.7, ≥ 0.7), migrant status (migration from outside the area between 2000 and 2006), and rainfall (total rainfall in past seven days <40 mm vs. ≥ 40 mm). We built univariate and multivariable proportional hazards models to examine the effects of travel time on mortality hazard. We included an age adjustment (indicator variables for 2-month age strata from 0 to 11 months and 6-month strata from 12 to 59 months) to control for the changing age distribution of the population over time and a calendar time adjustment (six-month time strata) to control for temporal trends. To account for spatial clustering of deaths in our models, we used a spatial bootstrap method with 50 repetitions. In each repetition, we randomly selected 40 sublocations (with replacement) and estimated the proportional hazards model on all data from the selected sublocations. Standard errors for regression coefficients were obtained as the standard deviation of coefficients across repetitions. Variables without statistically significant effects (at the 0.05 level) based on Wald tests were dropped from the multivariable models.

All data were double-entered into File Maker Pro 5.5 and cleaned using Stata 9.2 (StataCorp, College Station, TX). Analyses were conducted in Stata 9.2.

### Analysis age groups and timeframe

To ensure comparability with other demographic and epidemiological studies, the analysis was conducted for under-5 year olds, under-1 year olds and 1 to 4 year olds separately. We excluded data from the period during which death ascertainment was incomplete, restricting the analysis to 2004-6 for infants and to 2003-6 for children 1 to 4 years of age.

### Ethical approval

This study was approved by the Scientific Coordinating Committee and Ethical Review Committee of the Kenya Medical Research Institute and by the Institutional Review Board of the Johns Hopkins Bloomberg School of Public Health.

## Results

The analytic dataset included 93,216 children followed for 150,782 person-years. We observed 1599 under-5 deaths, 1125 under-1 deaths, and 734 1 to 4 year old deaths. Mortality rates were 13.2 per 1,000 child-years in under-5s (95% CI: 12.6-13.9), 46.0 per 1,000 in under-1s (95% CI 43.4-48.7) and 5.8 per 1,000 in 1 to 4 year olds (95% CI: 5.4-6.2). The under-5, infant, and child mortality ratios were 64.6 (95% CI: 61.6-67.7), 46.2 (95% CI: 43.6-48.9) and 21.8 (95% CI: 18.2-22.2) per 1,000 live-births, respectively.

### Spatial variations in mortality

**Figure 2:**
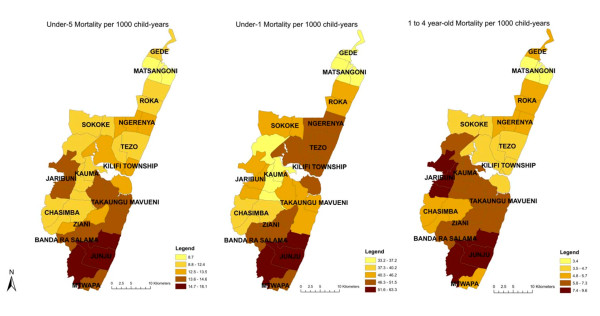
**Under-5, Under-1, and 1 to 4 year-old mortality by location in the Kilifi Epi-DSS**.

We first explored variations in mortality by administrative location (Figure 2). Under-5 mortality rates varied from 8.8 to 18.1 per 1,000 child-years across locations, under-1 rates from 34.5 to 61.9 per 1,000 and 1 to 4 year old rates from 3.4 to 9.7 per 1,000. Hazard ratios from Cox models comparing individual locations to Kilifi Township ranged from 0.69 to 1.37 in under-5s, from 0.69 to 1.05 in infants and from 0.73 to 2.06 in older children, and these differences were statistically significant (log-rank tests: p < 0.01). Spatial patterns in mortality differed across age groups. Kilifi Township and the surrounding areas to the north had high infant mortality, as did the most southern locations. In contrast, higher child mortality was observed in the western and southern parts of the Epi-DSS, but not in the Township or its northern neighbors.

**Figure 3:**
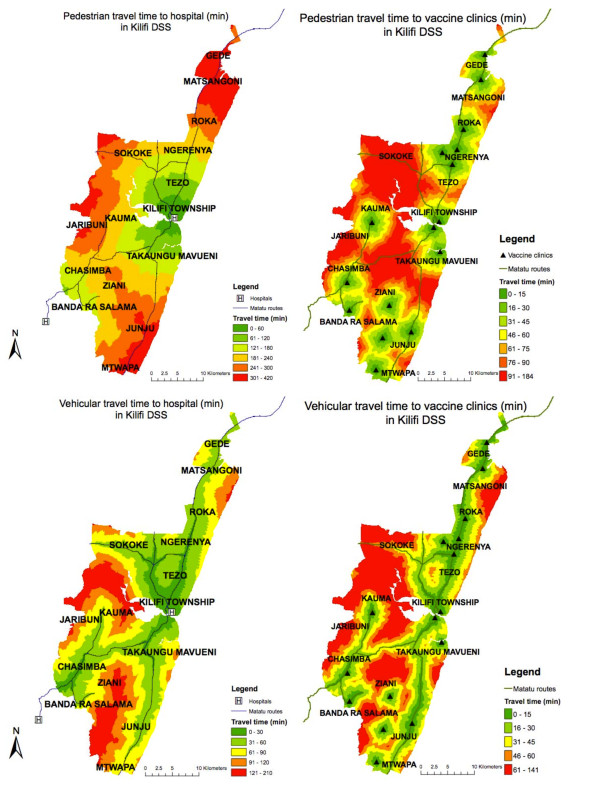
**Pedestrian and Vehicular Travel Times to Hospitals and Vaccine Clinics in the Kilifi Epi-DSS**.

Among children residing in the Epi-DSS, the median Euclidian distance to health facilities was 12.4 km for hospitals (inter-quartile range (IQR): 8.1-16.9 km) and 2.8 km for vaccine clinics (IQR: 1.5-4.7 km). Overall, 22% of children lived 5 kms' distance or more from a vaccine clinic and 0% lived 10 kms' distance or more away. Median pedestrian and vehicular travel times to hospital were 193 min (IQR: 125-267) and 49 min (32-72), respectively. Analogous values for vaccine clinics were 47 min (25-73) and 26 min (13-40) (Figure 3). In categorical analyses, there were no clear trends of increasing or decreasing mortality with increased pedestrian or vehicular travel time to hospital. We therefore constructed a log-linear model using continuous travel time variables, consistent with our original hypothesis (Table 1). Mortality hazard increased by 9% per 1/2 hour of vehicular travel time in children 1 to 4 years old (HR = 1.09, 95% CI: 1.03; 1.16). No associations were seen in under-1s or for pedestrian travel time. In the case of vaccine clinics, pedestrian travel time was not associated with mortality, whereas higher vehicular travel time was associated with lower mortality in infants (HR = 0.95 per 15 min, 95% CI 0.92-0.99).

**Table 1:** Univariate proportional-hazards models for mortality vs. travel time variables and covariates of interest in children under 5 years old, under 1 year old and 1 to 4 years old: hazard ratios and 95% confidence intervals

	<5 years			<1 year			1-4 years		
**Risk variable**	**HR**	**(95% CI)**	**p-val**	**HR**	**(95% CI)**	**p-val**	**HR**	**(95% CI)**	**p-val**

Travel time variables									
Hospital -- pedestrian (per 60 min)	0.99	(0.95,1.04)	0.73	0.98	(0.94,1.01)	0.32	1.02	(0.96,1.09)	0.47
Hospital -- vehicular (per 30 min)	1.01	(0.95,1.08)	0.73	0.98	(0.92,1.03)	0.40	**1.09	(1.03,1.16)	<0.01
Vaccine clinics -- pedestrian (per 15 min)	1.00	(0.98,1.03)	0.73	1.00	(0.97,1.03)	1.00	1.02	(0.98,1.06)	0.43
Vaccine clinics -- vehicular (per 15 min)	0.97	(0.93,1.02)	0.25	*0.95	(0.92,0.99)	0.03	1.02	(0.96,1.07)	0.58
Sex									
Female	1			1			1		
Male	**1.15	(1.04,1.28)	<0.01	**1.23	(1.08,1.40)	<0.01	1.01	(0.82,1.23)	0.96
Ethnicity			0.03			0.17			<0.01
Giriama	1			1			1		
Chonyi	1.04	(0.90,1.19)	0.62	1.00	(0.86,1.15)	0.95	1.15	(0.96,1.37)	0.14
Kauma	0.89	(0.76,1.04)	0.14	0.83	(0.68,1.02)	0.07	1.09	(0.88,1.35)	0.42
Luo	1.47	(0.97,2.23)	0.07	1.10	(0.01, >99)	0.99	**3.08	(1.83,5.17)	<0.01
Duruma	*1.56	(1.04,2.35)	0.03	1.64	(0.91,2.94)	0.10	*1.49	(1.03,2.15)	0.03
Jibana	1.28	(0.93,1.77)	0.13	1.18	(0.86,1.60)	0.31	1.15	(0.73,1.81)	0.54
"Other"	1.05	(0.81,1.36)	0.70	1.14	(0.93,1.41)	0.21	0.73	(0.49,1.09)	0.13
Proportion of women with any education			0.98			0.51			0.41
<0.5	1			1			1		
0.5-<0.6	0.96	(0.77,1.21)	0.75	1.05	(0.87,1.26)	0.61	0.84	(0.66,1.07)	0.10
0.6-<0.7	0.99	(0.81,1.22)	0.93	1.10	(0.86,1.40)	0.47	0.79	(0.51,1.21)	0.17
≥ 0.7	0.97	(0.80,1.17)	0.71	1.12	(0.96,1.31)	0.14	0.67	(0.40,1.13)	0.18
Migrant status									
Non-migrant	1			1			1		
Migrant	0.93	(0.82,1.06)	0.29	0.91	(0.75,1.10)	0.32	1.02	(0.77,1.35)	0.89
Rainfall									
< 40 mm per 7 days	1			1			1		
≥ 40 mm per 7 days	**0.71	(0.62,0.80)	<0.01	**0.72	(0.93,1.02)	<0.01	**0.69	(0.56,0.86)	<0.01

(*p < 0.05; ** p < 0.01)

Note: The analysis of maternal education was specified 'per location' not per mother.

We performed two sub-analyses that resulted in similar mortality patterns across locations and travel time strata as the primary analysis (data not shown). The first sub-analysis excluded Kilifi Township in order to consider only the rural area of the Epi-DSS. The second excluded all children whose mothers had migrated in the three months prior to their date of birth; this analysis aimed to correct for the possibility that women with high-risk pregnancies migrated from outlying areas into town in order to give birth at the hospital, which would inflate neonatal mortality ratios in the Township.

### Multivariable proportional-hazards models

Univariate Cox model results for each of the predictors we considered are shown in Table 1. The final model estimates appear in Table 2. Pedestrian travel times to hospital and vaccine clinics were not associated with death hazard in any of the age groups. The effect of vehicular travel time to hospital on mortality was similar to that seen in the univariate models, with a positive association in 1 to 4 year olds that achieved borderline statistical significance (HR = 1.08 per 1/2 hour of travel time, p = 0.06). In infants, mortality risk decreased with increasing vehicular travel time to vaccine clinics (HR = 0.96 per 1/4 hour, p = 0.01).

**Table 2:** Multivariable proportional-hazards models^1 ^for mortality vs. travel time to hospital and vaccine clinics in children under 5 years old, under 1 year old, and 1 to 4 years old: hazard ratios and 95% confidence intervals

Age	<1 year		1-4 years		<5 years	
Travel model	HR	95% CI	HR	95% CI	HR	95% CI
Time to hospital						
Pedestrian travel (per 60 min)	0.98	(0.95-1.02)	1.02	(0.96-1.08)	0.99	(0.95-1.04)
Vehicular travel (per 30 min)	0.98	(0.93-1.03)	*1.08	(0.99-1.17)	1.02	(0.95-1.08)

Time to vaccine clinics						
Pedestrian travel (per 15 min)	1.00	(0.98-1.03)	*1.02	(0.99-1.05)	1.02	(0.99-1.04)
Vehicular travel (per 15 min)	**0.96	(0.93-0.99)	1.02	(0.95-1.08)	0.98	(0.92-1.05)

(*p < 0.05; ** p < 0.01)

1. Final models included sex, ethnic group and rainfall and adjusted for age and calendar time. Significant effects are detailed in the Results section.

Boys had higher mortality than girls in any analysis that included young infants: hazard ratios ranged from 1.15 (p < 0.01) in under-5s to 1.23 in under-1s (p < 0.001) and 1.40 in under-2 month olds (p < 0.001), signifying that the increased hazard of death was concentrated in the neonatal period. Ethnicity was highly associated with mortality in under-5s and 1 to 4 year olds but not in infants. The hazard of death was more than three-fold higher in Luo children and approximately 1.5-fold higher in Duruma children 1 to 4 years of age than in Giriama children. In children under-5 overall, the increase in hazard was similar among the Luo (HR = 1.4-1.5, p = 0.04-0.07) and the Duruma (HR = 1.5-1.6, p = < 0.01 to p = 0.07). Mortality hazard was 17% lower in high rainfall than low rainfall periods in under-5s and under-1s (p = 0.02 to p = 0.05). Maternal education and migrant status were not associated with mortality hazard in any of the models, and there were no consistent, statistically significant interactions of any of the predictors with travel time.

## Discussion

In this study, we investigated the relationship between child mortality and physical access to health care in a low-income setting that has recently seen substantial investments in health infrastructure. We found two-fold variations in observed infant and under-5 mortality across the 900 km^2 ^Epi-DSS area. However, our data did not lend support to the widely held notion that mortality increases with distance to hospitals and vaccine clinics: in children 1 to 4 years old, mortality increased with vehicular travel time to hospitals; in infants under-1 year old, mortality decreased with increasing vehicular travel time to vaccine clinics; in each case the effects were small and no associations were observed in other age groups.

These findings contrast with published analyses from rural Tanzania [20], the Democratic Republic of Congo [22] and Burkina Faso [23] showing a strong relationship between mortality and distance to health facilities but are consistent with another DSS study from the Gambia [21]. In Burkina Faso and the DRC, the density of health facilities was significantly lower than in our setting, with 35% of families residing more than 10 km from the nearest clinic and 65% more than 5 km away, respectively, as compared to 0% more than 10 kms' and 22% more than 5 kms' distance in Kilifi. The study populations in Tanzania and the Gambia had relatively similar physical access to care to the population in Kilifi: in both studies, mortality increased with distance in univariate models; only the Gambia study presented a multivariable analysis in which this effect disappeared. In Kilifi, there was no relationship between travel time to hospital or vaccine clinics and mortality overall, but children living more than 2 hours by vehicle from the hospital had worse survival than those living less than 2 hours away. Together, these findings suggest that the high density of health services available in our study area may explain the lack of an association between travel time to health facilities and mortality. Because the Kilifi DSS is representative of Kenya as a whole in terms of physical access to health care with approximately two-thirds of the population within one hour's walk of a primary care facility [27], we expect these results to be generalizable to most of the country.

Several sources of bias and confounding may have influenced our results. First, methodological errors may have led us to inaccurately estimate mortality risk in this population. The lack of an association between travel time to hospitals and infant or under-5 mortality and the negative relationship between vehicular travel time to vaccine clinics and infant mortality were partly driven by a high hazard of death in Kilifi Township, which was concentrated in the early neonatal period. We conjectured that women with high-risk pregnancies may migrate from outlying areas into town in order to give birth at the hospital, leading to increased neonatal mortality in town. However, location-specific mortality and survival patterns did not change when we excluded children whose mothers had migrated in the three months prior to giving birth, suggesting that pregnancy-related migrations did not bias this analysis. While we cannot rule out other errors in data collection or cleaning procedures, these are unlikely to vary spatially and should therefore not affect our results.

Second, the assumptions underlying our travel time models may require refinement. Significant effects were seen for vehicular travel time to hospital in older children and for vehicular time to vaccine clinics in infants. Pedestrian and vehicular travel times to both hospitals and vaccine clinics were 70% correlated (Spearman's rank correlation rho = 0.69, p < 0.01). The absence of an effect for pedestrian travel time to hospital or vaccine clinics may reflect high levels of vehicular transport usage in the Epi-DSS. However, data from other Kenyan districts suggest that a majority of patients walk to the hospital, irrespective of distance [13,27]. The choice of transport mode may depend on a variety of considerations such as distance, availability of disposable income to cover matatu costs (from 20 to 120 Kenyan shillings, or US$0.26 to 1.56 per trip) and perceived severity of a child's illness. Further, even if theoretical travel times to the nearest clinic are accurate, they may not reflect actual travel times, as families are likely to use more distant clinics thought to provide higher quality services based on drug availability, staffing and other factors [27-29]. Detailed studies of matatu, bicycle, and private vehicle usage patterns as well as health facility choice and its relationship to service quality are necessary to improve upon the travel time models proposed here [30].

Third, we were unable to account for a number of possible confounders of the relationship between travel time and mortality. Data from the antenatal clinic at Kilifi District Hospital have shown an HIV prevalence of 5 to 7% over the past five years, but geographically stratified data are lacking. In most of Africa, HIV prevalence is highest in urban areas [31,32] and in close proximity to roads [33,34]. This could drive the high infant mortality rates in Kilifi Township and at shorter vehicular travel times to health facilities, negating or even inversing the effect of distance on mortality. Controlling for HIV in an individual-level analysis would require knowledge of the HIV status of all residents, since sublocation-level variables may mask heterogeneity within small areas; obtaining this information may not be feasible or ethical. Given the higher prevalence of HIV among immigrants from Western Kenya (primarily of Luo origin) than in the local population, adjusting for ethnicity may diminish but is unlikely to fully eliminate this source of confounding. Socio-economic data from the Epi-DSS area were not obtainable for individual residents, and we resorted to sublocation-level maternal education as a proxy variable. Maternal education has been shown to correlate highly with traditional measures of socio-economic status such as income and expenditures. However, we were unable to capture socio-economic inequalities within sublocations, which can be substantial (personal communication: C. Molyneux) and have a strong impact on mortality [9,10]. Finally, other spatial factors may confound the association between travel time to health facilities and mortality. Ecological features affect the risk of childhood infectious diseases, such as pneumonia, malaria or diarrhea [35]. Socio-behavioral characteristics determine adherence to various risk-reduction interventions, such as the use of insecticide-treated nets [18,36]. We were unable to correct for these and other sources of clustering in mortality risk in our models. Further analyses adjusting for these factors or stratified by cause-of-death should be conducted when additional individual-level data become available.

## Conclusions

In this study, we aimed to investigate the relationship between infant and child mortality and distance to hospitals and vaccine clinics, and had sufficient data to detect small trends with distance. The primary analysis showed no effect of travel time on mortality in children less than 5 years of age, though these results should be interpreted with caution in light of our inability to adjust for some important potential confounders. In our Epi-DSS, two-thirds of children live within one hour's walk of a government clinic, a situation representative of Kenya as a whole [27]; while mortality varies over small areas by a factor of nearly two-fold, physical access to health facilities explains little of this variation. These findings suggest that in countries like Kenya, where the time to reach health facilities for most children is not great, geographic access to health care is not a determinant of child mortality. With physical access targets nearly met, further improvements in child survival will only be achieved through renewed examination of the social, behavioral and quality-of-care factors that impede access to preventive and curative services.

## Competing interests

The authors declare that they have no competing interests.

## Authors' contributions

JCM, OSL and JAGS designed the study. JCM, HG and JAGS cleaned and analyzed the data. AMN developed geographic analysis methods. TNW and EB supervised data collection. All authors contributed to preparation of the final report. All authors read and approved the final manuscript.

## Pre-publication history

The pre-publication history for this paper can be accessed here:

http://www.biomedcentral.com/1471-2458/10/142/prepub
